# Ultrasound‐Induced Mechanoluminescence and Optical Thermometry Toward Stimulus‐Responsive Materials with Simultaneous Trigger Response and Read‐Out Functions

**DOI:** 10.1002/advs.202201631

**Published:** 2022-06-16

**Authors:** Yicong Ding, Byoungjin So, Jiangkun Cao, Lothar Wondraczek

**Affiliations:** ^1^ Otto Schott Institute of Materials Research Friedrich Schiller University Jena Fraunhoferstrasse 6 Jena 07743 Germany

**Keywords:** heating, mechanoluminescence, thermometry, ultrasonic

## Abstract

Ultrasound‐induced mechanoluminescence (USML) of Erbium‐doped CaZnOS is reported. Using the fluorescence intensity ratio of the ^2^H_11/2_, ^4^S_3/2_ → ^4^I_15/2_ transitions of Er^3+^ allows for simultaneous temperature mapping at an absolute sensitivity of 0.003 K^−1^ in the physiological regime. The combination of USML, local heating, and remote read‐out enables a feedback and response loop for highly controlled stimulation. It is found that ML is a result of direct energy transfer from the host material to Er^3+^, giving room for adapted spectral characteristics through bandgap modulation. ML saturation at high acoustic power enables independent control of local light emission and ultrasonic heating. Such USML materials may have profound implications for optogenetics, photodynamic therapy and other areas requiring local illumination, heating, and thermometry simultaneously.

## Graphical abstract

Using the fluorescence of Er^3+^ in a piezoelectric matrix allows for simultaneous ultrasound‐induced mechanoluminescence (USML), local heating, and optical temperature mapping at high absolute sensitivity. This combination of stimulus‐response and remote read‐out enables a feedback loop for highly controlled stimulation. Such USML materials may have profound implications for optogenetics, photodynamic therapy, and other areas requiring local illumination, heating, and thermometry simultaneously.

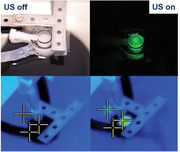

## 1 Introduction

Light emission by mechanical stimulation—termed mechanoluminescence (ML) —has been reported for a broad range of materials, from inorganic crystals and polycrystalline ceramics^[^ ^1^ ^]^ to organic and hybrid compounds.^[^ ^2^ ^]^ Correlations between the level of applied stress and the intensity of luminescence enable a variety of applications,^[^ ^3^, ^4^ ^]^ for example, in stress sensors, crack detectors, or smart displays.^[^ ^5^, ^6^, ^7^, ^8^, ^9^, ^10^ ^]^ ML induced by ultrasonic excitation (USML) was evaluated for the ability to visualize ultrasonic power distribution,^[^ ^11^, ^12^ ^]^ but also for security and anticounterfeiting purposes,^[^ ^13^ ^]^ or as a photon source in photocatalysis,^[^ ^14^ ^]^ photobioreactors,^[^ ^15^ ^]^ and optogenetics.^[^ ^16^ ^]^ In comparison to conventional ML excited by static or tactile forces, USML offers a remote trigger for luminescent light emission; however, it is currently much less exploited. Here, we use high‐intensity focused ultrasound (HIFU) as a noninvasive stimulus with the ability to penetrate hydrous media, biological tissue, or other materials, for USML excitation.^[^ ^16^ ^]^ Combining HIFU‐ML with the temperature‐sensitive ^2^H_11/2_, ^4^S_3/2_ → ^4^I_15/2_ transition of Er^3+^ in an Erbium‐doped compound enables simultaneous USML, ultrasonic heating, and optical thermometry with a single particle probe. This combines dual trigger response and read‐out functionality, for example, in photodynamic therapy.

## 2 Results and Discussion

### 2.1 Material Synthesis and Optical Properties

**Figure 1:**
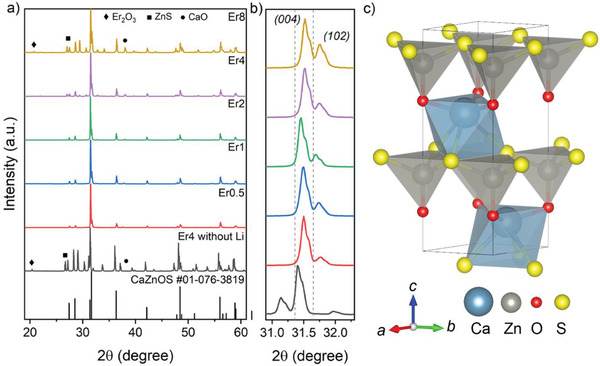
a) XRD patterns and b) zoom at the region of 2*θ* ≈ 31° … 32° of as‐prepared specimens of CaZnOS: Er^3+^ with and without the fluxing agent Li_2_CO_3_. The standard XRD pattern of CaZnOS is provided for reference (JCPDS #01‐076‐3819). c) Crystal structure of CaZnOS. Er^3+^ ions are expected to substitute the Ca^2+^ sites.^[^ ^18^ ^]^ The dashed lines in panel b) indicate the positions of the (004) and (102) reflexes in the reference diffraction data of CaZnOS.

We synthesized CaZnOS:*x*Er^3+^ with a dopant concentration of *x* = 0.5–8 mol%. A flux of 2 wt% of Li_2_CO_3_ facilitated the incorporation of Er^3+^ ions into the crystalline matrix of CaZnOS. Er4 (*x* = 4, without flux) exhibited the X‐ray diffraction pattern of CaZnOS (JCPDS #01‐076‐3819), together with unreacted ZnS (JCPDS #36‐1450) and CaO (JCPDS #70‐5490) (**Figure** **1a**). Additional traces of Er_2_O_3_ (JCPDS #43‐1007) in Er8, Er4, and in the Er4 sample without flux indicated the practical limit of Er^3+^ incorporation when using the present synthesis conditions. According to X‐ray diffraction (XRD) data, fluxed samples Er0.5 to Er2 were pure of any residual ZnS or Er_2_O_3_. The intensity of the (004) lattice plane (2*θ* ≈31.5°) was significantly enhanced over that of (012) (2*θ* ≈ 31.7°) (Figure 1b), indicating preferred crystal growth along the *a,b*‐axes as a result of the presence of Li_2_CO_3_ (what leads to platelet crystal morphology^[^ ^17^ ^]^).

CaZnOS exhibits a hexagonal structure with noncentrosymmetric P6_3_mc, thus, it is piezoelectric (Figure 1c). The cell parameters are *a* = *b* = 3.757 Å and *c* = 11.404 Å.^[^ ^19^ ^]^ The two cations form oxysulfide polyhedra (Zn^2+^: *CN* = 4, *r*_Zn_ = 0.60 Å, one oxygen, three sulfur; Ca^2+^: *CN* = 6, *r*_Ca_ = 1.00 Å, three oxygen, three sulfur) arranged in alternating layers; the two cation sites can accommodate a variety of transition metal (e.g., Mn^2+[^ ^20^ ^]^) or rare‐earth dopant species (e.g., Tb^3+^,^[^ ^21^ ^]^ Nd^3+[^ ^22^ ^]^), leading to a broad variety of ML characteristics. According to Figure 1b, the most‐intense diffraction peak of (004) shifts to higher angle with increasing Er^3+^ dopant concentration, as does the (102) peak, too. This indicates lattice contraction, caused by Er^3+^ being incorporated on octahedral Ca^2+^ sites (with *r*_Er_ = 0.89 Å).^[^ ^23^ ^]^ Further evidence for this substitution process was previously reported on the basis of X‐ray absorption spectroscopy.^[^ ^18^ ^]^

**Figure 2:**
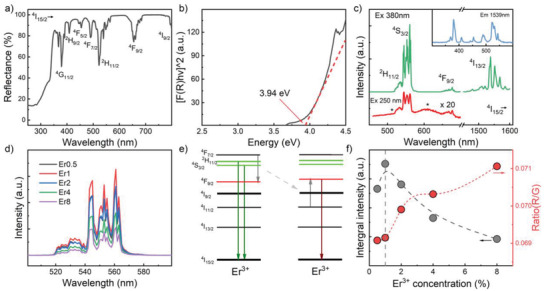
a) Diffuse reflection spectrum of CaZnOS: 1Er. b) Tauc plot of (*ahv*)^2^ versus *hv* derived from the diffuse reflection spectral data, and estimated bandgap energy. c) PL (*λ*_ex_  =  250 and 380 nm) and PLE (*λ*_em_  =  1539 nm) spectra of CaZnOS:1Er. d) PL spectra in the range of 500–600 nm for CaZnOS:*x*Er (*x* = 0.5, 1, 2, 4, and 8) at 380 nm excitation. e) Schematic energy‐level diagram showing cross relaxation between Er^3+^ ions. f) The integral intensity and ratio of *I*_red_ (^4^F_9/2_ → ^4^I_15/2_) and *I*_green_ (^2^H_11/2_, ^4^S_3/2_ → ^4^I_15/2_) for increasing Er^3+^ dopant concentration.

The diffuse reflection spectrum of the Er1 sample is shown in **Figure** 2a. It contains strong absorption bands, caused by the electronic transitions of ^4^I_15/2_ to ^4^G_11/2_ (380 nm), ^2^H_9/2_ (409 nm), ^4^F_5/2_ (454 nm), ^4^F_7/2_ (490 nm), ^4^H_11/2_ (522 nm), ^4^S_3/2_ (540 nm), ^4^F_9/2_ (654 nm) in Er^3+^. The band edge is in the range of 290–330 nm; we used the Tauc plot to estimate the electronic bandgap energy by extrapolating the linear fit of the edge‐region to *F*(*R*) = 0 using the Kubelka–Munk function, *F*(*R*) = *a*/*S* = (*1*‐*R*)^2^/(*2R*), where *a* is the absorption, *S* is the scattering coefficient, and *R* is the reflection (Figure 2b).^[^ ^24^ ^]^ This estimate provided a bandgap of 3.94 eV (≈315 nm), which is in accordance with previous report.^[^ ^25^ ^]^

Spectra of Er^3+^‐related photoluminescence (PL) were recorded at excitation wavelengths of 250 and 380 nm (Figure 2c). The major PL bands observed in this way were ^2^H_11/2_ → ^4^I_15/2_ (green), ^4^S_3/2_ → ^4^I_15/2_ (green), and ^4^F_9/2_ → ^4^I_15/2_ (red), and ^4^I_13/2_ → ^4^I_15/2_ (infrared, as labeled in Figure 2c; normalized spectra are provided in Figure S1, Supporting Information). Incorporation of Er^3+^ into the CaZnOS lattice led to distinct Stark splitting.

With increasing Er^3+^ content, the intensity of the red emission band *I*_red_ (^4^F_9/2_ → ^4^I_15/2_, 660 nm, marked in red in Figure 2e) is initially enhanced due to cross‐relaxation from ^4^F_7/2_ + ^4^I_11/2_ → ^4^F_9/2_ + ^4^F_9/2_ between neighboring Er^3+^ species. The maximum in PL emission intensity occurred at *x* = 1 for excitation at 380 nm (Figure 2d). For higher dopant concentration, concentration quenching occurred. This is also evident from the intensity ratio of *I*_red_ (^4^F_9/2_ → ^4^I_15/2_) and *I*_green_ (^2^H_11/2_, ^4^S_3/2_ → ^4^I_15/2_), *R/G*, which increased due to the relative increase of *I*_red_ (Figure 2f).

At the wavelength of 250 nm (4.96 eV), the excitation energy is higher than the bandgap energy of the lattice. Hence, host‐lattice excitation occurs. The overall band structure (peak positions) of the corresponding emission spectrum corresponds very well to that of direct excitation of Er^3+^:^4^I_15/2_ → ^4^G_11/2_ at 380 nm (3.26 eV, see Figure S1a, Supporting Information), however, we found a relative increase in the green and—even stronger—red bands, with broad background emissions located at around 520 and 610 nm. These background bands originate from the host‐lattice emission; they also occur in the undoped compound (Figure S2, Supporting Information). This is strong proof for lattice‐coupled excitation,^[^ ^25^, ^26^, ^27^, ^28^ ^]^ whereby the ^4^F_9/2_ level Er^3+^ is populated by energy transfer from the host lattice. This excitation and energy transfer path has profound consequences of the ML mechanism in CaZnOS materials.

### 2.2 Mechanoluminescence During Impact Loading

**Figure 3:**
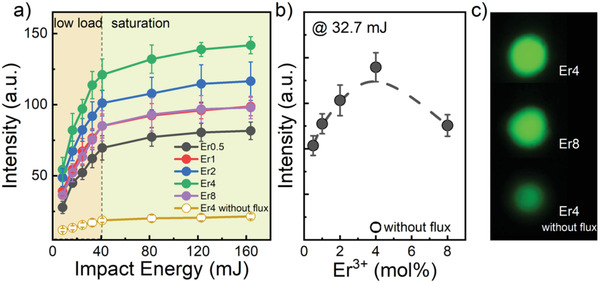
ML intensity from CaZnOS:Er during ball‐drop experiments as a function of a) impact energy (for variable Er^3+^ doping concentration) and b) Er^3+^ doping concentration (at a constant impact energy of 32.7 mJ). In c) photographs of visible impact ML are depicted (corresponding to the data shown in panel b)).

Impact‐ML was initially evaluated in ball‐drop experiments, whereby the drop‐height was used to adjust the impact energy in the range of 10–180 mJ (Figure **3**a). We found two regimes for the dependence of ML on impact energy, i.e., a first regime up to loads of about 40 mJ, where the ML intensity increases linearly with impact energy, and a saturation regime where the increase in ML levels‐off with further enhancement of the impact energy. A similar saturation phenomenon was discovered for ZnS:Mn.^[^ ^29^ ^]^ In general, the occurrence of a saturation regime is attributed to a limit in the charge density within the trap states.^[^ ^30^, ^31^, ^32^ ^]^ Furthermore, triboelectric effects affect the further increase of ML intensity.^[^ ^33^, ^34^ ^]^ We used an impact energy near the maximum of the low‐load regime in order to further evaluate the impact of Er concentration on ML performance (Figure 3b). Under this condition, the maximum ML intensity was observed for Er4 (*x* = 4 mol%), what is notably different from the onset of concentration quenching of PL (observed for *x* = 1 mol%, see Figure 2f). We attribute the origin of this difference to the nature of the dominant energy migration path for PL and impact‐ML, corroborating previous observations.^[^ ^35^, ^36^, ^37^, ^38^ ^]^ PL quenching originates from the energy transfer between Er^3+^ ions, whereas the quenching of impact‐ML is also affected by the presence of host‐lattice defects such as induced by Er^3+^ doping: in both cases, i.e., ML and PL, the Er^3+^ ions serve as luminescent centers, but their excitation occurs through variable paths. Furthermore, Er^3+^ also regulates the trap density, which determines the intensity of ML.^[^ ^39^ ^]^ Concentration quenching is a competitive process between the rate of depopulation of the active energy level, and the rate of carrier injection. The amount of injected carriers can be changed through changing the energy migration path. Concentration‐dependent PL spectra are provided in Figure S3 (Supporting Information) for 250 nm excitation. The intensity of the two broad bands around 520 and 610 nm decreases with increasing of Er^3+^ content, indicating that either the number of defect states decreased (which is not expected, since Er^3+^ doping facilitates defect formation), or that the rate of energy transfer from defects to Er^3+^ increased.

PL lifetime analyses corroborate this interpretation (Figures S4 and S5, Supporting Information). For the green emission excited at 380 nm, we observed a single‐exponential decay function with reduced lifetime for higher Er^3+^ concentration. When exciting at 250 nm, an additional, fast decay component is observed, which we attribute to emission from defect states partially overlapping with Er^3+^ emission. For the red emission, 380 nm excitation leads to slower decay, corroborating the occurrence of cross‐relaxation.

Noteworthy, a similar discrepancy of concentration quenching was observed in ZnS:Mn^2+^,^[^ ^40^ ^]^ CaZnOS:Mn^2+[^ ^41^ ^]^ or Er^3+^,^[^ ^21^ ^]^ and CaNb_2_O_6_:Pr^3+^.^[^ ^42^ ^]^

### 2.3 Ultrasound‐Induced Mechanoluminescence

We demonstrate that CaZnOS:Er^3+^ exhibits USML; USML can be exploited for multiresponsive devices with a remote trigger. **Figur** **e** **4** shows the set‐up we constructed for quantitative USML experimentation. As the excitation source, we used HIFU with an acoustic power of up to 40 W (at 293 K) (**Figure** 5a). For an excitation power of up to 10 W, the USML intensity increased linearly with a slope of ≈5 W^–1^ (*R* ^2^ = 0.95). At higher power, we observed a saturation effect, where the intensity did not increase any further with increasing acoustic excitation power (Figure 5b). This response function resembles the saturation behavior observed in the impact test; both may be due to limits in the achievable trap charge density.

**Figure 4:**
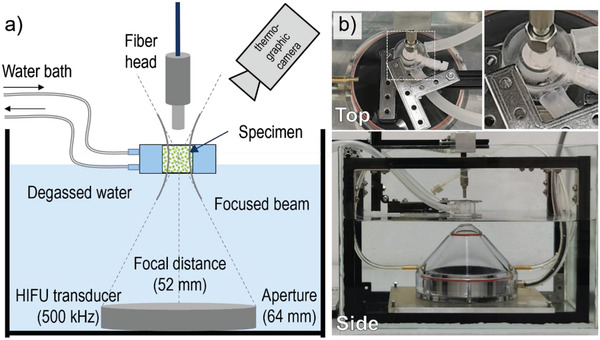
a) Schematic and b) photographs of the experimental set‐up used for quantitative HIFU‐induced mechanoluminescence.

**Figure 5:**
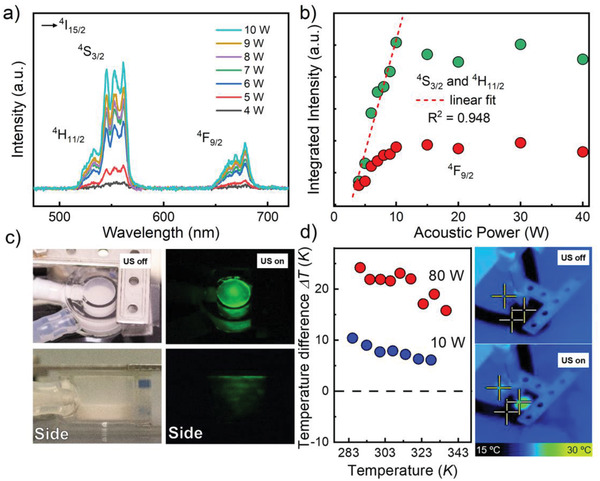
a) ML spectra obtained under variable ultrasonic acoustic power in the range of 4–10 W and b) integrated ML intensity of both emission regions (green: 500–600 nm; red: 625–700 nm) as a function of ultrasonic acoustic power. The dashed line in panel b) is from a linear data fit in the range of ≈4–10 W. c) Enlarged image of the peripheral ring‐shaped cuvette with cycling water. Specimen powders were located in the inner cavity; cycling water was used for regulating the sample temperature. The left‐side photographs were taken under ambient light with US off. Right‐side photographs in panel c) shows the HIFU‐induced ML in dark environment with US on. d) Temperature difference measured using a thermographic camera during HIFU sonication, and thermographic photos with ultrasound on and off.

### 2.4 Temperature Effect on PL and USML

Aside USML, HIFU can induce local heating in response to the acoustic pressure wave.^[^ ^43^ ^]^ For example, in the present set‐up using water as the immersion medium, irradiation in the range from 283 to 363 K yielded a local temperature increase of 7.80 K at 10 W acoustic power, and 20.73 K at 80 W (Figure 5d): at higher circulating temperature of the water bath, the temperature rise was less pronounced due to the increased heat loss to the surroundings. In principle, the temperature‐dependence of the Er^3+^ PL characteristics then enables direct monitoring of such a heating effect by optical thermometry.

**Figure 6:**
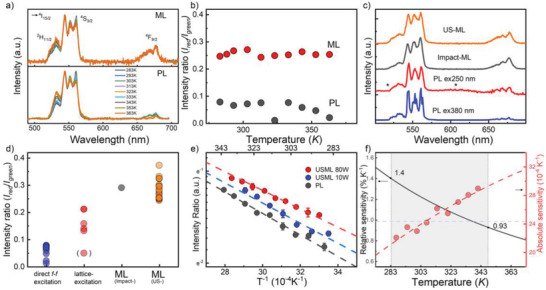
Normalized a) PL spectra under 369 nm excitation and ML spectra under HIFU excitation as a function of temperature for the corresponding samples. b) Integral intensity and ratio of *I*_red_ (^4^F_9/2_ → ^4^I_15/2_) and *I*_green_ (^2^H_11/2_, ^4^S_3/2_ → ^4^I_15/2_) for Er4 at temperatures from 283 to 363 K. c) Comparison of PL spectra at 380 nm excitation, 250 nm excitation, and ML spectrum under ultrasound sonication with a power of 10 W. d) Comparison of the intensity ratio of *I*_red_ (^4^F_9/2_ → ^4^I_15/2_) and *I*_green_ (^2^H_11/2_, ^4^S_3/2_ → ^4^I_15/2_) under different excitations. e) Intensity ratio of *I*_530nm_ and *I*_545nm_ from photoluminescence and mechanoluminescence, respectively, as a function of real temperature (then dashed lines in panel e) correspond to exponential data fits). The dashed‐lines are to guide the eye. f) Relative and absolute sensitivity versus temperature. The shaded‐region is for physiological regime.

**Figur** **e** **6**a depicts the temperature‐dependent, normalized PL and USML spectra over the range of 283–363 K, for an excitation wavelength of ≈369 nm and a HIFU acoustic power of 80 W, respectively. Assumedly, ML could be more susceptible to temperature variations as compared to PL because energy transfer to the dopants has to compete with additional energy dissipation channels in the host.^[^ ^44^ ^]^ The intensity ratio of the two green bands (^2^H_11/2_, ^4^S_3/2_ → ^4^I_15/2_) could provide temperature information; it will be evaluated later. The intensity ratio of the red (*I*_red_, ^4^F_9/2_ → ^4^I_15/2_) and the green band (*I*_green_, ^4^H_11/2_, ^4^S_3/2_ → ^4^I_15/2_) is also useful, because it is independent of thermal quenching and excitation intensity.^[^ ^28^, ^45^ ^]^ During PL under direct *f–f* excitation, this band ratio monotonically decreased with increasing temperature (except for an outlier at 323 K), whereas the USML intensity did not change significantly in the same temperature range (Figure 6b). The *I*_red_/*I*_green_ ratios obtained from different spectrometers (spectrofluorometer and fiber spectrometer) are similar, with differences lying within the error range of the employed equipment (less than 10%). The increase in temperature leads to an increase in lattice parameters and cell volume, and usually induces a spectral shift due to a decrease of the crystal field strength. The spectral shift has been observed for rare‐earth ion doping at high pressure as well as for transition metal ions;^[^ ^33^ ^]^ here, the increase in the Er^3+^ interatomic distance caused by lattice expansion reduces the cross‐relaxation probability, which then facilitates population of the ^2^H_11/2_ level as compared to that of ^4^F_9/2_ (leading to reduced *I*_red_/*I*_green_). However, in USML, this is further affected by interactions between the Er^3+^ dopant and the host lattice. In this case, the energy transfer probability may increase with temperature, what may compensate the reduction of cross‐relaxation (on a side note, we exclude lattice variations due to mechanical compression^[^ ^46^ ^]^ from our discussion because the excitation intensity remained unchanged).

The origin of ML is often associated with piezoelectrically‐detrapped carriers from a trap state, triggered by lattice strain. Then, the released electrons migrate directly to the excited state of the active luminescent center,^[^ ^33^, ^47^ ^]^ where radiative relaxation occurs. Alternatively, indirect migration is possible through the conduction band.^[^ ^41^, ^48^ ^]^ The discrepancy of PL and ML spectra, *i.e*., a spectral shift^[^ ^49^ ^]^ or the absence of high‐energy emission bands,^[^ ^42^ ^]^ is used as a qualitative indicator of these reactions. Thereby, the fluorescence‐intensity ratio can be a probe for the excitation paths^[^ ^28^ ^]^ because it is sensitive to the interatomic distance of Er^3+^ species, to host lattice interactions, and to the energy distribution in the pertinent (electronic) structure. Under direct excitation (380 nm, ^4^I_15/2_ → ^4^G_11/2_), the emission spectrum is primarily composed of a strong green component and a minor red one (Figure 6c). Because of nonradiative relaxation of the ^4^F_9/2_ level to lower‐lying levels, the red emission remains weak. Indirect excitation using band transition from the host lattice (250 nm) is relatively less efficient due to the additional requirement of host‐to‐dopant energy transfer; in this case, we find a strong green band, a weaker red one, and further, broader emission envelopes near 500 and 612 nm. These latter bands are associated with oxygen vacancies,^[^ ^25^, ^33^ ^]^ indicating the existence of multiple localized trap states.^[^ ^25^ ^]^ As shown in Figure 6d, the ratio of *I*_red_/*I*_green_ doubles from direct to host lattice excitation. This indicates very different relative populations of the ^2^H_11/2_, ^4^S_3/2_, and ^4^F_9/2_ levels between PL and ML. The spectral consistency between the absorption of ^4^F_7/2_ (490 nm), ^4^H_11/2_ (522 nm), and ^4^F_9/2_ states (654 nm) and the transitions from the trap states suggest the presence of energy transfer though the conduction band as well as of direct resonant energy transfer from the host lattice to the ^4^F_9/2_ state, as well as to ^4^F_7/2_^[^ ^50^ ^]^ and ^4^H_11/2_ of Er^3+^; indirect excitation is similar to electroluminescence in that it shares the same reaction path. Under excitation by haptic impact or by ultrasonication, the ratio of *I*_red_/*I*_green_ increases further (Figure 6d). The magnitude of this additional change cannot be understood on the basis of mechanical strain alone.^[^ ^46^ ^]^ Instead, it could involve more efficient direct energy transfer from the host lattice to the ^4^F_7/2_ and ^4^F_9/2_ levels, eventually caused by a tunneling effect, or by direct energy transfer. Finally, we observed that impact and ultrasonication yielded similar values of *I*_red_/*I*_green_. This suggests that ML from different mechanical stimuli (such as friction, compression, and ultrasonic excitation) is caused through the same host lattice energy transfer process to Er^3+^ in CaZnOS.

### 2.5 Simultaneous USML and Optical Thermometry

The value of *I*_530nm_/*I*_545nm_ depends on the temperature in the vicinity of the luminescent probe, which in turn depends on the intensity of acoustic excitation and the surrounding bath temperature (Figure 6e). Combining HIFU and USML thus enables local heating, light emission, and optical thermometry through a single, remote stimulus. Thereby, the HIFU acoustic power enables independent tuning of the US heating effect. For demonstration, we use sample Er4 which had the strongest USML intensity. The temperature response was recorded as a function of HIFU stimulation using the fluorescence intensity ratio (FIR) of the 530 nm (*I*_530nm_, ^2^H_11/2_ → ^4^I_15/2_) to 545 nm (*I*_545nm_, ^4^S_3/2_ → ^4^I_15/2_) emission. The ^2^H_11/2_ and ^4^S_3/2_ levels of the Er^3+^ ion are thermally coupled; their separating energy gap is below 1000 cm^−1^. The emission intensity represent the population of each level; the intensity ratio increases with increasing temperature according to a Boltzmann distribution of the transition probability. Similar thermal coupling was previously observed in Sm^3+^ (^4^F_3/2_, ^4^G_5/2_ → ^4^I_9/2_)^[^ ^51^ ^]^ and Nd^3+^ (^4^F_3/2_, ^4^F_5/2_ → ^4^I_9/2_),^[^ ^52^ ^]^ which could be used as alternative dopant species, for example, in order to adjust the spectral range of optical thermometry. As present, we focus on Er^3+^ because there, the two emission bands do not overlap. Hence, it provides the strongest sensitivity for optical thermometry.^[^ ^45^ ^]^ The intensity ratio can be expressed as^[^ ^45^ ^]^

(1) $$R_{\mathrm{PL}}=\frac{I_{530\mathrm{nm}}}{I_{545\mathrm{nm}}}=C\cdot \exp\left(\frac{-\Delta E}{k_{B}T}\right)$$

where *R*_PL_ is the fluorescence intensity ratio, *I*_530nm_, and *I*_545nm_ are the luminescence intensities of the corresponding thermally coupled energy levels of Er^3+^, Δ*E* is the effective energy gap between ^2^H_11/2_ and ^4^S_3/2_; *k*_B_ is the Boltzmann constant, and *T* is the absolute temperature. *C* is an empirical correction factor representing the degeneracy of the state and the spontaneous emission probability. The *R*_PL_ was well‐fitted using Equation (1) in linearized form (*R* ^2^ = 0.989) with *C* = 7.99 (vs a reported value of 7.09^[^ ^50^ ^]^) (Figure 6e). For the energy gap Δ*E*, we obtained ≈780 cm^−1^, which compares‐well to the value 775 cm^−1^ found from the excitation spectra (Figure 2a).

For the USML intensity ratio, local heating by the sonication mentioned above had to be considered as an additional factor (Figure 6e). As a result of local heating, we assume a temperature rise of Δ*T*

(2) $$R_{\mathrm{ML}}=C\text{exp}\left[-\Delta E/k_{B}\left(T+\Delta T\right)\right]$$

Δ*T* results in a horizontal shift of the *R*(*T*) curve (Figure 6e); again, it can be obtained from the linearized data fit. For 10 W acoustic power, at which the USML intensity in the linear region was at its maximum (Figure 5b), it is Δ*T*_10W_ = 9.5 ± 1 *K* (*R* ^2^ = 0.974). At 80 W excitation (when the USML intensity was in the regime of saturation), Δ*T*_80W_ = 20.9 ± 0.6 *K* (*R* ^2^ = 0.986). These values are in good agreement with the values independently measured using a thermographic camera (7.80 and 20.73 K, Figure 5d, respectively), given the limits of spatial resolution of such direct observation.

The temperature‐sensing accuracy of USML is quantified using absolute sensitivity *S*_A_ and relative sensitivity *S*_R_, which are defined as the absolute and relative variation of the emission ratio *R* with temperature^[^ ^53^ ^]^

(3) $$S_{A}=\frac{\partial R}{\partial T}=R\cdot \frac{\Delta E}{k_{B}T^{2}}$$

(4) $$S_{R}=\frac{1}{R}\cdot \frac{\partial R}{\partial T}=\frac{\Delta E}{k_{B}T^{2}}$$

The corresponding absolute sensitivity *S*_A_ and relative sensitivity *S*_R_ data are provided in Figure 6f. In the temperature detection range of our experiment (≈287–341 K), the maximum *S*_R_ = 1.36% K^−1^ at 287 K, and the maximum *S*_A_ = 0.0029 K^−1^ at 341 K. Theoretically, *S*_R_ monotonically decreases as the temperature increases, whereas *S*_A_ reaches its maximum value of 0.0038 K^−1^ at 562 K. It is higher than 1% K^−1^, which is practically difficult to achieve near room temperature, especially in the physiological temperature rage (303–348 K).^[^ ^52^ ^]^ The value of *S*_A_ from mechanoluminescence is close to the values acquired from up‐conversion of other reported materials, such as 0.0062 K^−1^ of ZnO:Er^3+[^ ^54^ ^]^ and 0.0075 K^−1^ of LiNbO_3_:Er^3+^, Yb^3+^.^[^ ^55^ ^]^ This comparison clearly shows that optical thermometry that exploits mechanoluminescence has similar sensing capability as traditional optical thermometric methods, and also shows promising applications in real‐time observation during ultrasonic treatment. The method can also use other rare earth ions such as Nd^3+^ and Tm^3+^ that emit in the biological windows (650–950, 1000–1350, and 1500–1750 nm). More importantly, it provides a novel means for remote monitoring, for example, as an alternative to magnetic resonance thermometry.^[^ ^56^ ^]^ Using USML as a remote stimulus, local heating and light emission can be simultaneously triggered. The HIFU acoustic power allows to decouple light emission intensity from the extent of local heating, making use of ML saturation, in the present case, at an acoustic power greater than 10 W.

## 3 Conclusion

Er^3+^‐activated multiresponsive CaZnOS polycrystals were prepared and used for in situ local heating, illumination, temperature sensing, and mechanical imaging. Temperature and stress were detected simultaneously in a simple testing set‐up. Temperature‐dependent and acoustic‐power‐dependent USML were demonstrated. Comparison of the ratio of *I*_red_ (^4^F_9/2_ → ^4^I_15/2_) and *I*_green_ (^2^H_11/2_, ^4^S_3/2_ → ^4^I_15/2_) according to the different excitation methods revealed that PL and ML are governed by different energy‐transfer processes. ML had the same origin, regardless of the stimulation method. For optical thermometry using HIFU as the excitation source, a maximum relative sensitivity *S*_R_ = 1.36% K^−1^, and a maximum absolute sensitivity *S*_A_ *=* 0.029 K^−1^ were achieved in the temperature range of ≈287–341 K. We demonstrate that mechanoluminescence induced by ultrasound can be used as light source for optical thermometry, and thereby advance the design of a multiresponse sensors with heating function, for example, for applications from photochemical material synthesis and photocatalysis to biomedical imaging and photodynamic therapy.

## 4 Experimental Section

### Phosphor Synthesis

A series of Er‐doped CaZnOS phosphors with a nominal chemical composition of Ca_1−_ *_x_* ZnOS:(*x*/2)Er_2_O_3_ with an additional 2 wt% Li_2_CO_3_ as fluxing agent (*x* = 0.5, 1, 2, 4, and 8) was prepared using a high‐temperature solid‐state reaction procedure. For this, stoichiometric mixtures of CaCO_3_ (99.5%, Carl Roth), ZnS (99.99%, Acros Organics), Er_2_O_3_ (99.9%, Projector GmbH), and Li_2_CO_3_ (99.999%, Carl Roth) were homogeneously mixed by wet‐milling for 24 h in ethanol using a ball mill. The obtained slurries were dried and subsequently sintered in a covered alumina crucible at 1050 °C for 4 h in a horizontal tube furnace under N_2_ atmosphere with flow rate of 1.5 L min^−1^. The samples obtained from this procedure were ground in an agate mortar to yield a fine powder. Samples are coded as “Er*x*” where *x* = 0.5–8 is mol% Er. Further reference samples were produced without Li_2_CO_3_.

### Characterization

XRD patterns were acquired using an X‐ray diffractometer (MiniFlex 600, Rigaku) with Cu *K*_*α*_ radiation (*λ* = 1.54 059 Å). Diffuse reflection spectra were recorded using a double‐beam UV–vis–NIR spectrophotometer (Cary 5000, Agilent) over the spectral range of 250–800 nm. Photoluminescence (PL) and photoluminescence excitation (PLE) spectra were collected using a high‐resolution spectrofluorometer (Fluorolog‐3, Horiba) with a 450 W Xe lamp as the excitation source, and a 50 W Xe flash lamp for dynamic spectroscopy, respectively. An integrating sphere was used to obtain the PL intensity.

For impact‐mechanoluminescence (impact‐ML), 0.1 g sample of powder was homogeneously spread at the bottom of the sample holder with 20 mm inner diameter to form a thin layer. A stainless‐steel ball with a diameter of 16 mm and a mass of 16.705 g was then dropped vertically onto the powder using a guiding pipe. For the bottom of the sample holder, a transparent epoxy was used which also served as a window so that the impact‐induced ML could be observed on the sample backside. For this, ML light passing the window was reflected by a 45° mirror to a DSLR camera (D610, Nikon) or alternatively a CMOS camera (DCC1545M, Thorlabs) placed close to the window (the images shown in Figure 3c are photographs taken in this way). The exposure time was set to 3 s. Recorded images were converted to grayscale data, and the maximum grayscale values were used to evaluate the intensity of the impact‐ML. Before each ball drop test, the sample powder was irradiated using a 365 nm UV lamp of 5 W for 20 s. If not otherwise stated, each experiment involved 10 individual ball drops, for which the average and the standard deviation of the impact‐ML intensity were calculated.

Impact‐ML and USML studies were conducted using a compact fiber spectrometer (Shamrock 163, Andor) equipped with a CCD camera (iDus 420, Andor) for spectra acquisition. The blaze wavelength was the same as that of the PL system, i.e., 500 nm. Ultrasonic excitation was provided by a high‐intensity focused ultrasound (HIFU) transducer (H‐104, SonicConcepts). A water bath (RM6, Lauda) was used to control the temperature around the specimen. For this, specimens were placed in a peripheral ring‐shaped cuvette under water cycling between 283 and 363 K, which in turn was placed into the focal region of the HIFU transducer. The transducer was placed at the bottom of a tank filled with degassed water. The fiber head of the spectrometer used for collecting ML light was aligned close to the center of the cuvette. For USML experiments, specimens were initially illuminated (charged) with a 365 nm UV lamp for 30 s. Subsequently, specimens were kept in the dark for 60 s to allow for the decay of any PL afterglow. If not otherwise stated, the HIFU transducer was switched on for 20 s at 500 kHz with a duty cycle of 95% and an output power within 4–80 W. The duty cycle indicates the net time during which the pulsed HIFU is operative relative to the set time of operation. A thermographic camera (VarioCAM, Jenoptik) was used to independently monitor the changes of temperature in the environment of the HIFU focus region. In addition, PL spectra were also collected for comparison in same environment under the illumination of a UV lamp.

## Conflict of Interest

The authors declare no conflict of interest.

## Supporting information

Supporting Information

## Data Availability

The data that support the findings of this study are available from the corresponding author upon reasonable request.
