# Supplementary material for: Ultrasound‐Induced Mechanoluminescence and Optical Thermometry Toward Stimulus‐Responsive Materials with Simultaneous Trigger Response and Read‐Out Functions
Source: Adv Sci (Weinh). 2022 Jun 16;9(23):2201631. doi: 10.1002/advs.202201631 (PMC9376836; doi:10.1002/advs.202201631)
Supplement: Supplementary file 1 — Supporting Information [file ADVS-9-2201631-s001.pdf]

ADVANCED SCIENCE
Open Access

Supporting Information

for *Adv. Sci.*, DOI 10.1002/advs.202201631

Ultrasound-Induced Mechanoluminescence and Optical Thermometry Toward Stimulus-Responsive Materials with Simultaneous Trigger Response and Read-Out Functions

*Yicong Ding, Byoungjin So, Jiangkun Cao and Lothar Wondraczek**

Supporting Information

Ultrasound-induced mechanoluminescence and optical thermometry toward stimulus-responsive materials with simultaneous trigger response and read-out functions

*Yicong Ding, Byoungjin So, Jiangkun Cao, Lothar Wondraczek**

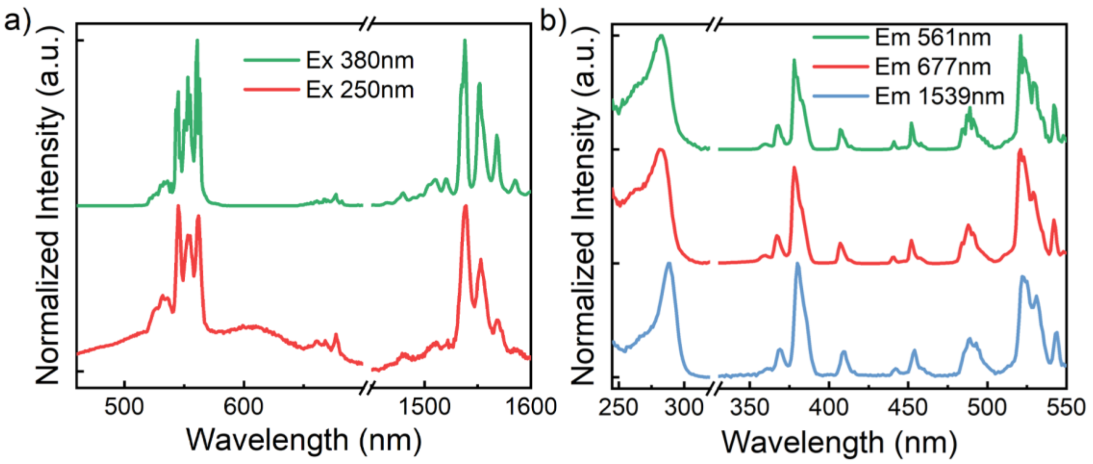

Figure S1. a) Normalized PL spectra at 250 nm and 380 nm excitation. b) Normalized PLE spectra at 561 nm, 677 nm and 1539 nm emission. All spectra are for the Er1 sample.

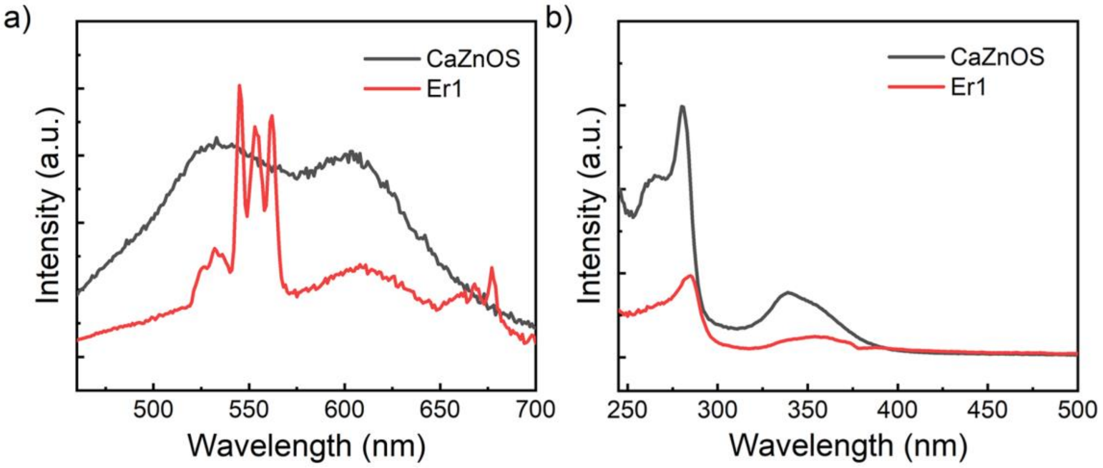

Figure S2. Photoluminescence a) emission spectra at 250 nm excitation and b) excitation spectra under 610 nm emission of undoped CaZnOS and Er1 sample.

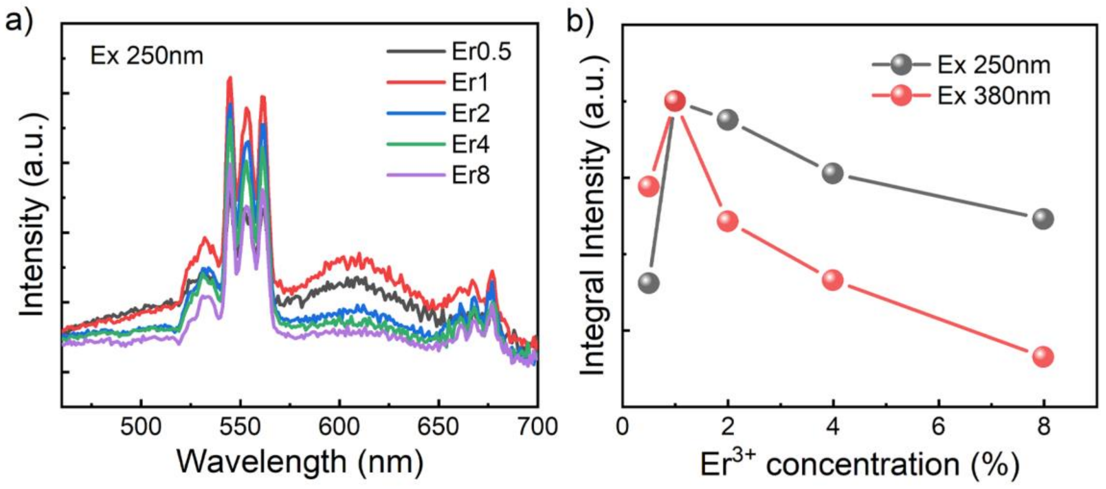

Figure S3. a) PL spectra in the range of 500 - 700 nm for CaZnOS:*x*Er (*x* = 0.5, 1, 2, 4 and 8) at 250 nm excitation. b) Comparison of integral intensity of the green emission ($^2H_{11/2}$, $^4S_{3/2}$ → $^4I_{15/2}$) for increasing Er^{3+} doping concentration with direct (380 nm) and indirect (250 nm) excitation.

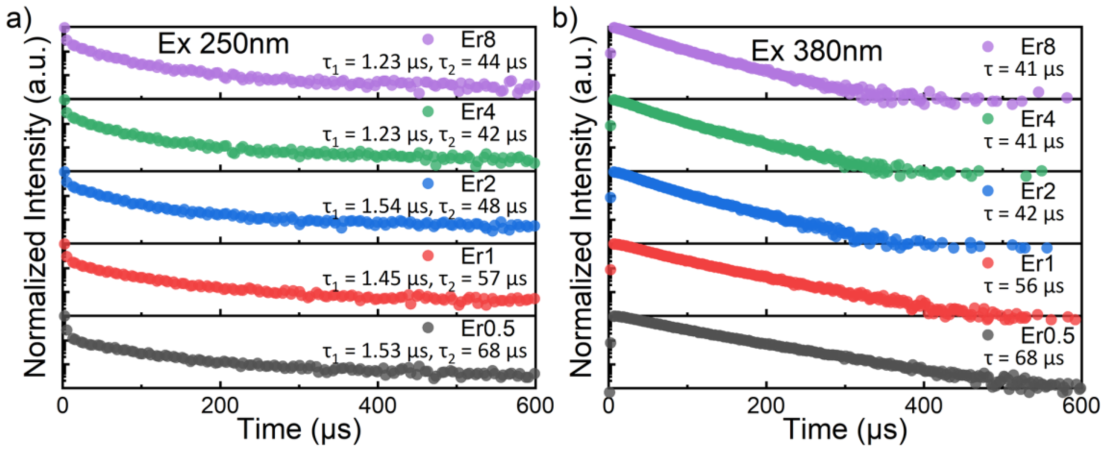

Figure S4. Fluorescence decay and estimated lifetime data for CaZnOS:*x*Er (*x* = 0.5, 1, 2, 4 and 8) at a) 250 nm excitation and b) 380 nm excitation, following the green emission band (561nm, $^4S_{3/2}$ → $^4I_{15/2}$).

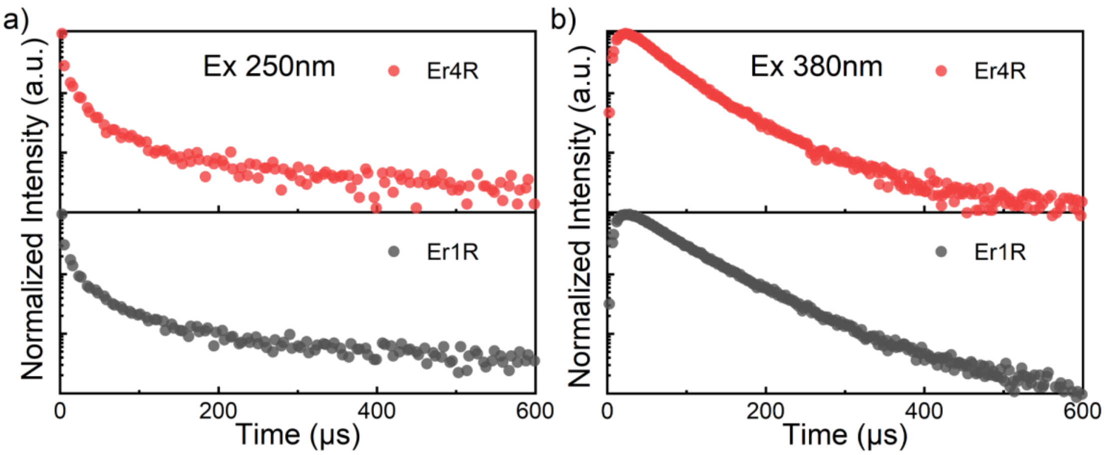

Figure S5. Fluorescence decay curves for samples Er1 and Er4 at a) 250 nm excitation and b) 380 nm excitation, following the red emission band (674nm, $^4F_{9/2} \rightarrow {}^4I_{15/2}$).

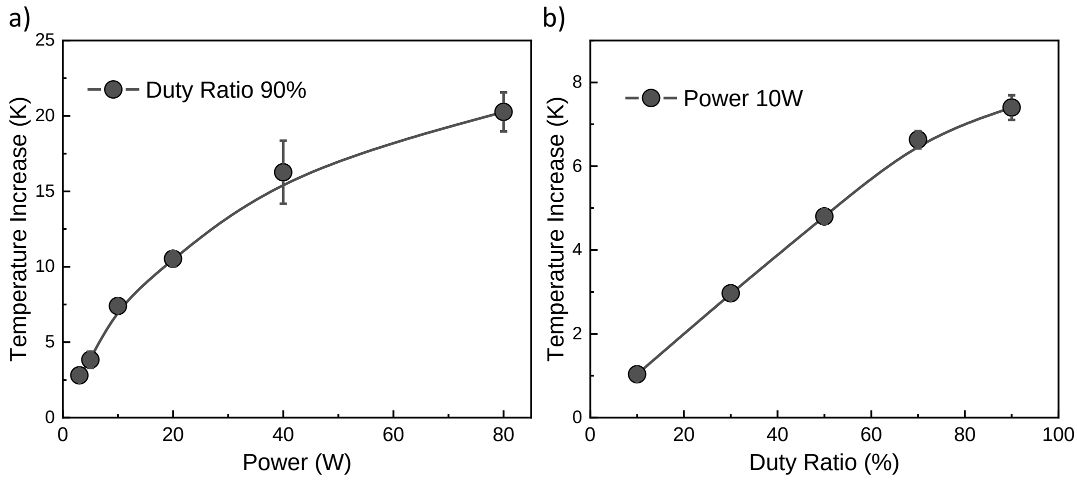

Figure S6. HIFU-induced temperature increase as recorded using a thermographic camera for variable acoustic power ((a), at constant duty ratio) and variable duty ratio ((b), at constant acoustic power).
